# Crashworthiness Design for Bionic Bumper Structures Inspired by Cattail and Bamboo

**DOI:** 10.1155/2017/5894938

**Published:** 2017-10-08

**Authors:** Tao Xu, Nian Liu, Zhenglei Yu, Tianshuang Xu, Meng Zou

**Affiliations:** ^1^School of Mechanical Science and Engineering, Jilin University, Changchun, China; ^2^Key Lab of Bionic Engineering, Ministry of Education, Jilin University, Changchun, China

## Abstract

Many materials in nature exhibit excellent mechanical properties. In this study, we evaluated the bionic bumper structure models by using nonlinear finite element (FE) simulations for their crashworthiness under full-size impact loading. The structure contained the structural characteristics of cattail and bamboo. The results indicated that the bionic design enhances the specific energy absorption (SEA) of the bumper. The numerical results showed that the bionic cross-beam and bionic box of the bionic bumper have a significant effect on the crashworthiness of the structure. The crush deformation of bionic cross-beam and box bumper model was reduced by 33.33%, and the total weight was reduced by 44.44%. As the energy absorption capacity under lateral impact, the bionic design can be used in the future bumper body.

## 1. Introduction

**Figure 1:**
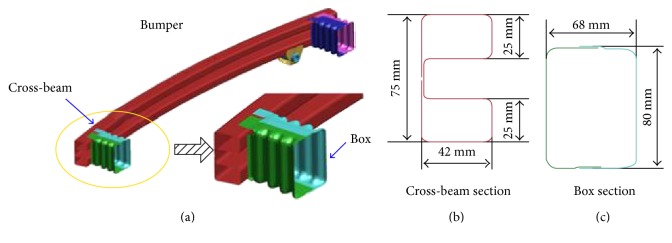
The FE model of bumper: (a) detail with enlarged scale, (b) the section of cross-beam, and (c) the section of crash box.

In traffic accidents, the bumper, side-door beam, and B-pillar of a car can absorb the impact of energy to ensure the safety of drivers and passengers. The bumper suffers from a lateral impact loading during the impact process. The primary function of a bumper is to attenuate the effects of a collision in direct contact [1]. Typically, the bumper of a car consists of four different parts: the fascia, the cross-beam, the crash box, and the crushable column [2]. The cross-beam under the bumper is usually loaded as a thin-walled section of the lateral compression (Figure 1). A lot of research has been carried out on the thin-walled structure in the process of straightening the horizontal compression [3]. The thin-walled structures are used as a good energy absorber in experimental investigation, analysis, and numerical methods.

Liu [4] had investigated numerically the conventional polygonal thin-walled columns with rectangular, octagonal, and curved hexagonal columns, in both quasi-static axial and lateral load conditions. Ahmad and Thambiratnam [5] had found that the conical tube filled with foam had better energy absorption performance than the empty conical tube. Under the axis compression conditions, Fan et al. [6] studied the hexagonal, octagonal, 12-sided, and 16-sided tubes experimentally and numerically. The results pointed out that the number of corners were directly related to energy absorption. Within a certain range, an increase in number of corners of the thin-walled column could help to improve energy absorption. Shen et al. [7] had studied the lateral crushing behavior of two concentric aluminum tubes of different diameters which were filled with aluminum foam. Fang et al. [8] had investigated the energy absorption characteristics of the functionally graded foam-filler into rectangular columns in transverse impact loading.

Some researcher had introduced the special cross sections of thin-walled structures as energy absorbers under various load conditions [9–11]. Loughlan et al. [12] had determined the coupled local distortions of thin-walled channel segments using finite element model. Alavi Nia and Parsapour [13] had investigated the mechanical behavior of the triangular, square, hexagonal, and octagonal sections of the thin-walled tubes under the quasi-static axial loading. Under quasi-static lateral loading, Baroutaji et al. [14] used the response surface and optimization methods to solve thin-walled rectangular tube energy absorption problem.

The thin wall structure studies in the abovementioned literature has a simple cross section. The complex cross sections of the thin-walled structures may have better crashworthiness than existing ones, but it remains an open challenge for researchers how to design a thin-walled structure with better crash performance and ease of production. After billions of years of evolution, some biological structures already have excellent properties and ingenious frameworks, which can provide inspiration to thin-walled structure designers.

Nowadays, bionic structures have gained the attention of researchers due to their excellent crash performance and very light weight. Bamboo [15, 16], horsetails [17, 18], and cattail [19] are kinds of gradient composite material with good mechanical properties in the natural environment. Due to bamboo's low density, it has a higher stiffness-mass ratio than some metallic materials such as steel and aluminum. The structural characteristics of horsetails lie in the cylindrical multi-cell structure which has good bending resistance. The emergent leaves exhibited a high slenderness ratio and a distinct twisting chiral morphology. It was found that the leaves have evolved multiscale structures and superior mechanical properties, both of which feature functionally gradient variations, to improve their resistance to failure. Various studies related to macrostructures and microstructures had shown that the gradient distribution of the plant has excellent mechanical properties [20–23]. Bionic structure could have better crashworthiness than traditional thin-walled structure when it is under the lateral dynamic loading. However, to the best of our knowledge, there are few studies on the crashworthiness of bionic structure under the condition of lateral dynamic loading [24–26].

In this work, two kinds of bionic structures was imitated: the structural characteristics of bamboo and cattail. We created a finite element (FE) model for the impact and validated our results with experiments. We simulated the process of energy absorption under axial/lateral loading and drop impact by using nonlinear finite element code LS-DYNA. The results showed that the design of bionic structure could be further improved for better crashworthiness and structural behaviors.

## 2. Bionic Design

In the nature, the biological structure need to adapt the surrounding environment. Many structures are lightweight along with good mechanical properties which can transport water and nutrients from root to leaf [27].

### 2.1. Cattail and Bamboo Plant

**Figure 2:**
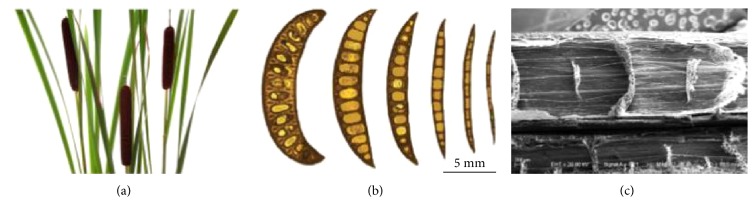
Cattail plants: (a) plants in the wild, (b) cross sections of an emergent leaf from the base to the apex, and (c) microstructures of an emergent leaf observed using SEM [19].

Cattail is also called Typha, which is an herbaceous perennial emergent aquatic macrophyte (Figure 2). They mainly inhabit streams, lakes, marshes, rivers, reservoirs, ditches, freshwater ponds, canals, and other shallow water areas [28, 29]. Due to its wide distribution and properties, the stems and leaves can be used as raw materials for candle cores, paper, ropes, and woven fabrics.

In order to understand the influence of chirality on cattail's mechanical behavior, the researchers investigated the twisted chiral morphology and the wind adaptation of banana leaves by experiment. From Figure 2(c), their multiscale structures had been observed using optical microscope, which have superior mechanical properties. It was found that the leaves have evolved multiscale structures and superior mechanical properties, both of which feature functionally gradient variations, to improve their ability of lodging resistance. The synergistic effect of chiral morphology and reconfiguration could greatly improve the survivability of cattail plants in the wind [19].

**Figure 3:**
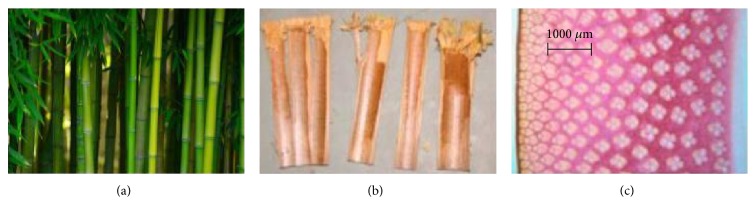
Bamboo: (a) collection site of bamboo, (b) internode samples, and (c) cross section [15, 16].

Bamboo is a typical tubular structure with good mechanical properties in the natural environment. They have a multilayered composite structure from the cellular level to the tissue level [30] (Figure 3). The excellent mechanical properties of bamboo depends on the hollowness, tubular shape, discrete distribution of the nodes, gradient distribution of the vascular bundles, and multilayer structure of the chondrocytes [20]. A large number of voids between the organizations make bamboo prone to splitting during axial loading which results in a reduction in the energy absorption of bamboo.

In this work, the cattail was used to design the bionic cross-beam structure and bamboo was the prototype of the bionic energy box.

### 2.2. Bionic Cross-Beam and Bionic Energy Box Design

**Figure 4:**
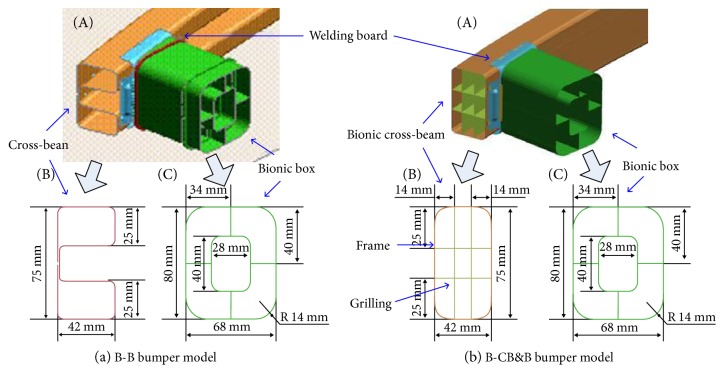
The bionic bumper models: (a) B-B bumper model and (b) B-CB&B bumper model.

In Figure 2(b), the internal rib structure of cattail can be a very good resistance to external pressure. According to its structural and functional characteristics, a bionic cross-beam with the structure of the cappuccino was designed (Figure 4(b), (B)).

In Figure 3(c), we showed the macroscopic structures of bamboo. The load was delivered by vascular bundles in the bamboo, which were connected with ground tissue (serving as a matrix). Therefore, the structure and function of these matrices were applied to the bionic structure to improve the load transfer and energy absorption efficiency. A bionic matrix designed to connect bionic cross-beam and transport loads is called a bionic box (Figure 4(a), (C)).

Bamboo has excellent bending and compressive properties, preventing the cracking phenomenon during the vertical and horizontal compression loading [25]. Hence, a welding plate was designed as a bionic node reinforcement rib for a bionic bumper structure.

Based on the relationship between the structure and the dynamic mechanical properties of cattail and bamboo, three components of bionic structures (i.e., bionic cross-beam, bionic box, and welding board) were designed. Two bionic bumper models, namely, bio-box bumper (B-B bumper) and bio-cross-beam and bio-box bumper (B-CB&B bumper) were developed by combining the different bionic components (Figure 4). The parameters of the bionic cross-beam, bionic box, and welding board are shown in Figure 4, and the structural parameters of the bionic structure are listed in Table 1. Bionic cross-beam and bionic box adopted the extrusion aluminum instead of high-strength steel to reduce the total weight. The materials were all the standard types which can be purchased from the suppliers.

## 3. Numerical Model

### 3.1. Structural Crashworthiness Criteria

Properly defined crash performance standards are critical to the evaluation of the crash bionic bumper. The widely use concept in collision standards are energy absorption (EA, kJ), mean crushing force (MCF, N), and specific energy absorption (SEA, kJ/N). SEA denotes the energy absorbed per unit mass of the absorber, which is often used to estimate the energy absorption capabilities of the structures.

(1) $$\begin{array}{c} SAE=\frac{EA}{M}, \end{array}$$

where *M* represents the total mass of the structure. EA represents the energy absorption in crash, which can be formulated as

(2) $$\begin{array}{c} EA=\int_{0}^{s}F\left(x\right)dx, \end{array}$$

where *s* represents the crash displacement and *F* denotes the impact force. From (2), it can be seen that the SEA value is higher for the design of the energy absorption structure that has better energy absorption capability in the collision process.

### 3.2. FE Modeling Method

**Figure 5:**
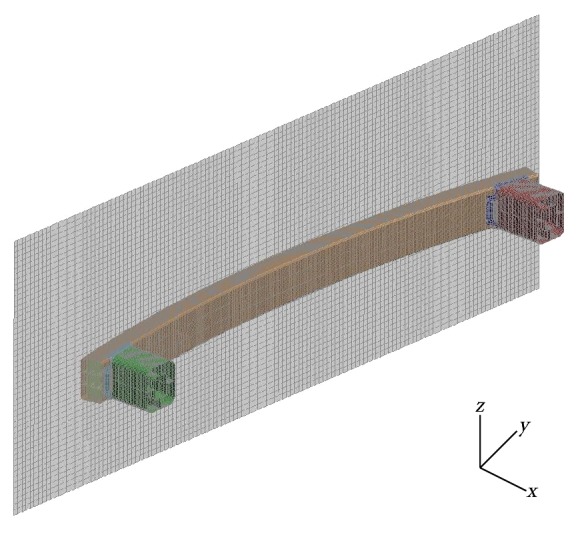
The meshing of the B-B bumper FE model.

The finite element models were developed using Hypermesh 14.0, and the collision analysis was carried out using commercial code LS-DYNA. Shell elements were used to set the wall. In this work, the material failure of aluminum alloy tube was not considered. It was not necessary that the smaller grid size would significantly improve the accuracy of the simulation results, but it could definitely increase the computation cost. Thus, before the tests, the accuracy of the mesh was investigated. We tested force versus displacement for five different mesh sizes and adopted the element size of 2.0 × 2.0 mm in all our simulations. The FE model of one of the B-B bumper under lateral impact was shown in Figure 5. There were several materials such as high-strength steel and extrusion aluminum that had been used as the wall in different applications.

**Figure 6:**
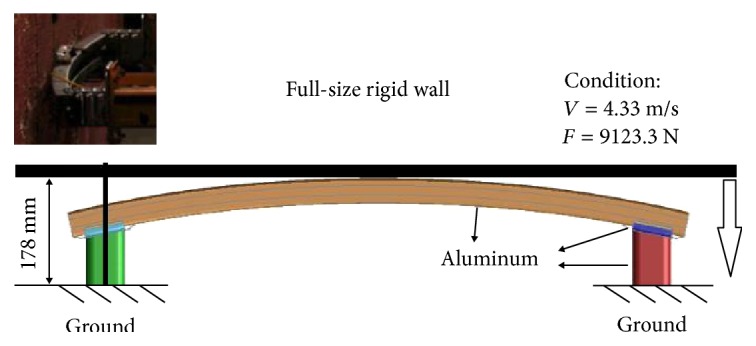
The loading condition of the full-size impact simulation.

In this work, simulation had been performed to analyze the energy absorption effect of the bionic bumpers. It was a positive impact test, in which we used a full-size rigid wall to crush the bumper model that resembles a car in a frontal collision. All three models have been impacted by a rigid wall, and the height of the rigid wall in the impact tests was converted to the real collision. By calculating kinetic energy conservation, the full-size rigid wall's weight was 9123.3 N. The initial speed of the test was 4.33 m/s. The loading of the test had been shown in Figure 6, and the material of components were listed in Table 1.

## 4. Numerical Simulation and Analysis

### 4.1. Validation of the FE Model

Frontal crash test was one of the main tests to verify the passive safety performance of vehicles [31]. The bumper of a car was tested under the condition of a frontal crash. In this experiment, the initial bumper impacted onto the rigid wall at 4.4 m/s. To validate the FE models, the bumper model with the same geometry, shape, and loading condition as the experiment was developed and simulated. Thus, the experimental results validated the finite element model in this study.

**Figure 7:**
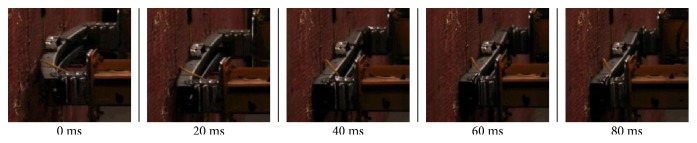
Full-size crash test of initial bumper by experiment.

**Figure 8:**
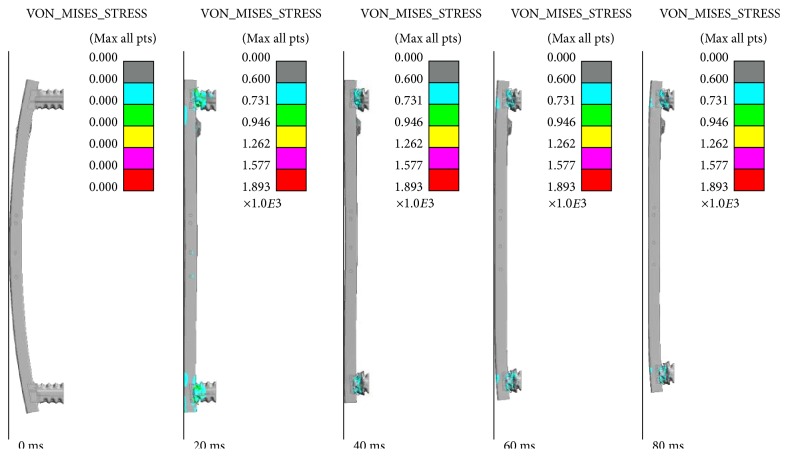
Full-size crash test of initial bumper by simulation.

In Figure 7, we showed the distortion of bumper during the collision process, which was shot by high-speed camera. From 0 to 40 ms, cross-beam absorbed impact energy and produced large plastic deformation. From 40 to 60 ms, the collision energy was transmitted to the box which produced plastic deformation. After 60 ms, the structure was destroyed by the impact which could not absorb any impact energy.  Figure 8 displayed the FE model's comparison of crash behavior by simulation. From Figures 7 and 8, it could be seen that the simulation results were in close agreement with experimental results. Therefore, FE simulations could be used reliably to study the behavior of the bionic bumpers.

### 4.2. Full-Size Crash Simulation

**Figure 9:**
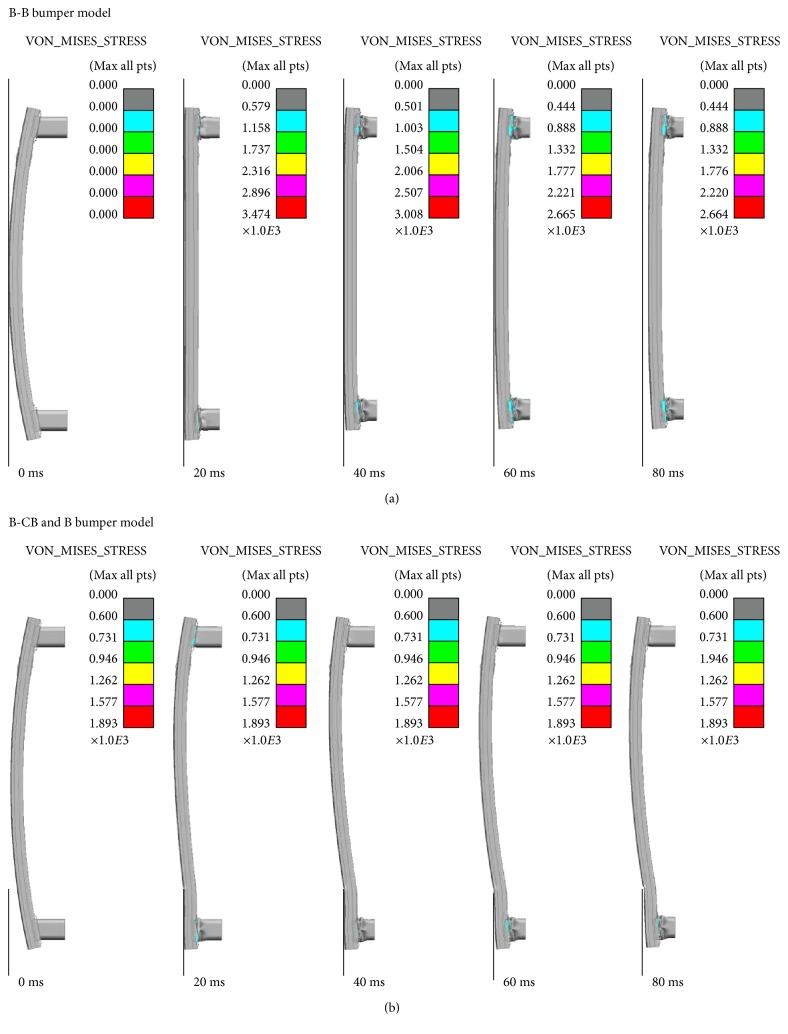
Full-size crash test of two bionic bumper models by simulation: (a) the result of B-B bumper model and (b) the result of B-CB&B bumper model.

In materials science and engineering, the von Mises stress is a scalar value of stress which can be computed from the Cauchy stress tensor [32]. It is used to predict yielding of materials under complex loading. In this case, the crushing and deformation process of the two bionic bumper structures were shown in Figure 9. From the figure, it could be seen that the absorption box had contact with the cross-beam and thus had higher Mises stress and therefore folded progressively in 20 ms. Compared with the initial bumper model, the distribution of Mises stress in two kinds of bionic structure bumper models was smaller and the crushing phenomenon had happened after 40 ms. In the B-CB&B bumper model, the bionic cross-beam also had the distribution of Mises stress, which showed that the bionic cross-beam had played the role in energy absorption. The folding patterns of the bionic cross-beam and box had played an important role in the full-size crash simulation of the new bionic bumper structure. According to the structures and mechanical behavior of the three models, the bionic cross-beam and bionic box were the main components to effectively improve the stability of the models on defending the failure of loads.

**Figure 10:**
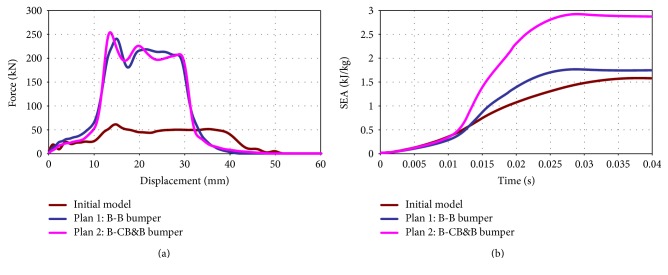
Crashworthiness characteristics associated with full-size crash to the bumper models: (a) lead-displacement curve and (b) SEA-time curve.

Figure 10 showed the load-displacement and SEA-time curves, respectively, for the three types of bumper models under full-size crush loading. The curve of the initial bumper was lower than that of the bionic bumper structures. The peak of the B-CB&B's curve was higher than the peak of the B-B bumper model. The results of energy absorption, deformation, and total weight were shown in Table 2. Compared with the initial bumper model, the energy absorption of B-B bumper model and B-CB&B bumper model was increased by 15.16% and 83.03%, respectively. And the crush deformation was reduced by 35.90% and 33.33%. The mechanical properties and deformation behavior of the samples indicate that the internal core structure had a great influence on the mechanical properties of the models. The separation of the cross wall with a lightweight core increased the moment of inertia of sandwich structures that could make the structure to resist the bending and buckling loads. The energy absorption of the two bionic bumper models was higher than the initial bumper model, and the deformation of the two bionic models was much smaller than the initial one [15, 16]. Comparing the two bionic bumper models, it could be seen that the B-CB&B model with multimaterial had the best energy absorption characteristics with the least weight. Inspired by the gradient distribution of cattail and bamboo, bionic cross-beam structure had compression performance and bionic energy box had bending characteristics. These results showed that the bionic structure can effectively improve the impact resistance of bumper structure.

## 5. Conclusion

In this study, we had investigated two bionic bumper models under full-size crash and size impact loading, with both bumpers imitating the structural characteristics of cattail and bamboo. Three FE models had been investigated on crashworthiness using nonlinear finite element code LS-DYNA. According to the numerical results, the bionic bumper models had remarkably effected their crashworthiness. The main points concluded from this work are as follows.

1. Inspired by the gradient distribution of cattail and bamboo, two bionic bumper structure models had been designed, which were composed of a bionic cross-beam, a bionic box, and a welding board. The bionic cross-beam served the same function as that of the inner structure of the cattail, the bionic box acted as the matrices and vascular bundles of bamboo, and the welding board resembled the bamboo's joint.
2. The results showed that the bionic bumper models had better energy absorption characteristics than the initial model. The BCB&B bumper had the best energy absorption characteristics. Meanwhile, the crush deformation was reduced by 33.33% and the total weight was reduced by 44.44% for the bionic bumper model.
3. The numerical results showed that the bionic bumper structure exhibited potential advantage of energy-absorbing capabilities under conditions of full-size impact. However, the bionic structure needs further studies to explore the complex structure of cattail and bamboo. The bionic structure can be used as an energy absorber in vehicle bodies and other engineering applications.

## Figures and Tables

**Table 1:** The parameters of models.

Model	Component	Part	Material	Thickness (mm)
Initial bumper	Cross-beam		High-strength steel (trip800)	1.8
	Box		High-strength steel (st280)	1.8
B-B bumper	Cross-beam		High-strength steel (trip800)	1.8
	Box		Extrusion aluminum (6062T6)	1.5
	Welding plate		High-strength steel (st280)	1.4
B-B&CB bumper	Cross-beam	Frame	Extrusion aluminum (6062T6)	2.0
		Grilling	Extrusion aluminum (6062T6)	1.7
	Box		Extrusion aluminum (6062T6)	1.5
	Welding plate		High-strength steel (st280)	1.4

**Table 2:** Results.

Model	Energy absorption (kJ/N)	Deformation (mm)	Total weight (N)
Initial bumper	1.550	39	52.974
B-B bumper	1.785	25	49.05
B-CB&B bumper	2.837	26	29.43
